# Levosimendan use in critical care - a case series

**DOI:** 10.1186/2197-425X-3-S1-A540

**Published:** 2015-10-01

**Authors:** C Gibson, R Appelboam

**Affiliations:** Royal Devon and Exeter Hospital, Intensive Care Medicine, Exeter, United Kingdom

## Introduction

Levosimendan is a calcium sensitiser that enhances myocardial contractility without increasing myocardial oxygen consumption or adversely affecting diastolic function. Whilst theoretically attractive, lack of outcome data in critically ill patients means that it is not yet established in intensive care practice. We have been using levosimendan on our Intensive Care Unit (ICU) for more than 6 years and the data presented here represents one of the largest case series on the use of levosimendan in this setting.

## Objectives

To retrospectively analyse the cases of patients on a general ICU who were treated with levosimendan.

## Methods

Our electronic patient record was used to identify patients who had been treated with levosimendan between 2009 and 2014. Patients' notes were analysed for demographic and clinical data. Subgroups for each answer were predefined and the best fit answer was decided by the investigator. A second search (2011-2013) was used to identify 2 matched control populations: acute myocardial infarction (AMI) and pneumonia. A chi-squared test was used to see if there was a difference in ICU outcomes between the matched controls and those treated with levosimendan.

## Results

Over the 5 year period levosimendan was used in 90 patient cases. Exactly half the patients died in the ICU (Table 1).

**Table:** Table 1

	ICU survivors (Mean (range))	ICU non-survivors (Mean (range))
No. patients	45	45
Age	61 (28-83)	71 (17-89)
APACHE-2 score	21 (8-36)	20 (0-37)

72% of patients had cardiac monitoring to aid diagnosis and response to treatment; 28% were treated on clinical grounds alone.

81% of patients were mechanically ventilated and 77% were already on vasoactive infusions.

3 patients were documented to have aortic stenosis, a contraindication to levosimendan.

33% of patients had a >20% fall in systolic blood pressure associated with the use of levosimendan. 6% had a new arrhythmia.

For patients with cardiac failure associated with AMI there was a statistically significant difference between the ICU mortality of those treated with or without levosimendan, favouring levosimendan. There were insufficient data to draw conclusions about those with pneumonia (Table 3).

**Table 2:** Indications for the use of levosimendan.

	No. cases	
Indication	ICU survivors	ICU non-survivors
Ischaemic heart failure	26	25
Sepsis induced cardiomyopathy	10	14
Acutely decompensated chronic heart failure	6	5
Right heart failure	1	3

**Table:** Table 3

	Control	Levosimendan	p-value
**Pneumonia**			
APACHE-2 score	23.7	23.9	
ICU mortality	41/168 = 24%	5/9 = 56%	N/A
**AMI**			
APACHE-2 score	16.7	19.8	
ICU mortality	47/78 = 60%	25/51 = 49%	p = 0.0247

## Conclusions

In this case series, the most common indication for levosimendan use was heart failure secondary to AMI. We observed an ICU survival benefit in using levosimendan in this group of patients. This finding is supported by the RUSSLAN study that showed a short-term survival benefit to using levosimendan in this patient group [1]. Whilst there is, to date, no evidence for a long-term mortality benefit in patients with AMI induced cardiac failure, there is supporting evidence for patients with acute decompensation of their chronic cardiac insufficiency [2].
